# Effect of the Built Environment on the Cumulative Incidence of Acute Diarrheal Diseases: A Community-Based Cohort Study From Underprivileged Areas of Bhopal, India

**DOI:** 10.7759/cureus.55942

**Published:** 2024-03-11

**Authors:** Yogesh Sabde, Uday K Mandal, Vikas Yadav, Tanwi Trushna, Rajnarayan R Tiwari

**Affiliations:** 1 Environmental Health and Epidemiology, Indian Council of Medical Research-National Institute for Research in Environmental Health, Bhopal, IND; 2 Epidemiology and Public Health, Indian Council of Medical Research-National Institute for Research in Environmental Health, Bhopal, IND

**Keywords:** diarrhea, infections, children, india, built environment

## Abstract

Background

Diarrhea is a major public health problem in under-five children worldwide. Various sociodemographic, environmental, and behavioral factors play a role in the occurrence of diarrheal disease in children under the age of five. This study aims to estimate the cumulative incidence of acute diarrheal diseases during a one-year study period and examine its association with the built environment factors among children under the age of five in selected underprivileged areas of Bhopal.

Methodology

We conducted this study in Bhopal, a city in central India. We surveyed the underprivileged dwellers of Vajpayee Nagar, Sanjay Nagar, and Mother India Colony of Bhopal city. This is a prospective cohort study with a one-year follow-up period to examine the cumulative incidence of acute diarrheal diseases among under-five-year-old children in the study area. Data were analyzed using SPSS version 25 (IBM Corp., Armonk, NY, USA).

Results

Data were collected from February 2021 to February 2022. A total of 658 families of eligible children were contacted to participate in the study. After excluding 38 participants for various reasons (denied consent: 3; lost to follow-up: 32; moved out of the study area: 3), data were finally analyzed for 620 participants. In the study among the under-five-year-old children, the cumulative incidence of acute diarrheal diseases was 23.8% (148 out of 620). In our study, lower age (adjusted odds ratio (OR) = 0.86, 95% confidence interval (CI) = 0.75-0.99, p = 0.041) and non-availability of flush latrine in the house (adjusted OR = 4.95, 95% CI = 1.80-13.59, p = 0.002) were statistically associated with a higher incidence of acute diarrheal disease among the study population.

Conclusions

In our investigation, we observed a cumulative incidence of diarrhea at 23.8% (148 out of 620) among the underprivileged under-five population residing in Bhopal, India. This incidence exhibited significant associations with younger age and the absence of in-house flush latrines.

## Introduction

Diarrhea is a prominent cause of mortality and morbidity in children under the age of five globally [1]. In 2017 alone, around 533,768 children under five years old died from diarrhea globally, accounting for 9.9% of all under-five childhood deaths [1]. According to the United Nations Children’s Fund, approximately 9% of deaths in children under the age of five years result from diarrhea, highlighting a concerning global trend [2]. Additionally, diarrhea significantly contributes to malnutrition, which is also a leading cause of mortality among under-five-year-old children, particularly in developing countries [3].

In developing countries, a child under five experiences diarrhea, on average, thrice a year [4,5]. Five nations, namely, India, Nigeria, Congo, Pakistan, and China, jointly contribute to half of the diarrhea-related deaths among children [5]. The National Family Health Survey (NFHS-5, 2019-2020) in India reported a diarrhea prevalence of 7.3% among children aged under five [6].

Numerous sociodemographic, environmental, and behavioral factors contribute to the prevalence of diarrheal disease among children under the age of five [7, 8]. Key sociodemographic factors encompass the child’s age, place of residence, maternal education, and household economic status [7-10]. Environmental factors, such as drinking water quality, sanitation facilities, waste disposal, and dwelling characteristics, are also linked to childhood diarrhea [7-10]. Additionally, behavioral factors, including breastfeeding practices, dietary habits, and handwashing practices, play a significant role in diarrhea among young children [7-10].

The built environment encompasses many factors, including housing conditions, sanitation facilities, water supply systems, and neighborhood infrastructure [11]. Previous research has established a link between these elements and health outcomes [12,13]. Still, a nuanced understanding of their collective influence on the cumulative incidence of acute diarrheal diseases needs to be improved, particularly in the context of marginalized communities. By employing a community-based cohort study design, in this study, we seek to overcome these knowledge gaps and provide evidence-based insights that can inform targeted interventions. Thus, the objective of this study was to assess the cumulative incidence of acute diarrheal diseases over the one-year study period and to investigate its association with built environment factors among under-five children in selected underprivileged areas of Bhopal.

## Materials and methods

This study is part of a broader investigation that seeks to assess the influence of housing conditions on the health of children aged under five in urban settings. The detailed protocol for this overarching research has been previously published, serving as the foundational framework for this study [14]. In this study, we are presenting the effects of the built environment on the cumulative incidence of acute diarrheal disease.

We conducted this study in Bhopal, which is the capital city of Madhya Pradesh, India. According to the 2011 census, Bhopal had a population of 2.4 million [15]. In 2005, the Indian government launched the Basic Services to the Urban Poor (BSUP) as a sub-mission within the framework of the Jawaharlal Nehru National Urban Renewal Mission [16]. The aim was to furnish essential services to the urban underprivileged population. A total of 19 such colonies were constructed to relocate underprivileged populations from slums to BSUP colonies in Bhopal [17]. There are also 0.4 million slum dwellers living in Bhopal [18]. To conduct this survey among the underprivileged population, we selected Vajpayee Nagar (BSUP colony), Sanjay Nagar (slum), and Mother India Colony (slum) based on their large population size and geographical proximity to each other.

This study adopted a prospective cohort design with a one-year follow-up period to examine the cumulative incidence of acute diarrheal diseases among under-five-year-old children in underprivileged areas of Bhopal. Follow-up visits were conducted monthly over a one-year duration to monitor and record the occurrence and frequency of acute diarrheal episodes among children aged under five.

For the analysis in this study, the inclusion criteria were that the family of the participant under five years old had been living in the same house for the last year. Exclusion criteria were families not providing consent, cases lost to follow-up, families moved out of the study area, known cases of congenital anomalies, moderate or severe cases of intellectual disability, children to a single parent, children of migrants or tenants or paying guest families, and family size more than six.

We used formula 4PQ/L^2^, where P is the prevalence of disease in previous research, Q is 1-prevalence, and L is the required precision of the estimate. Considering p = 25.2 (proportion) from previous research and 20% relative precision, the estimated sample size was 283 [19]. The original study was conducted with a sample size of 620 to compare the health status of under-five-year-old children living in BSUP (N = 299) and slum colonies (N = 321), which is higher than the required sample size [14]. Therefore, it can also be used to measure the cumulative incidence of diarrhea in a given area.

We retrieved the list of children residing in study areas from their respective Anganwadis. Anganwadis are community outreach centers under the Integrated Child Development Scheme in India, which maintains a list of under-five children for nutritional and educational services [20]. We selected the required number of children with simple random sampling. We recruited the eligible children after obtaining written informed parental consent. If two children were chosen from the same house, the child selected first was retained, and the second child was dropped and replaced by another child with the following random number from the list.

Data was collected by trained data-collecting staff using a pretested semi-structured questionnaire. During the first visit, questions were asked regarding family and demographic information. Subsequently, during monthly follow-up visits over 12 months, relevant medical history related to acute diarrheal disease in the last month was recorded by a trained pediatrician. For this study, we operationally defined diarrhea according to the definition provided by the Integrated Disease Surveillance Project of the government of India, i.e., “Passage of 3 or more loose watery stools in the past 24 hours with or without vomiting” [21].

During follow-up visits, concurrent environmental assessments were also conducted, concentrating on factors contributing to diarrheal diseases. These assessments encompassed the evaluation of water and sanitation facilities, the availability and quality of water sources, sanitation facilities, and family hygiene practices. Furthermore, the study delved into waste management, scrutinizing waste disposal practices and cleanliness in the proximity of households. Additionally, the assessment extended to identifying and evaluating areas with the potential for contamination, such as open sewage and waste dumps, within the neighborhoods of the study participants.

Data were collected using an open-access online data collection platform (i.e., ona.io) and then exported into SPSS Statistical Software version 25 (IBM Corp., Armonk, NY, USA). A child reporting at least one episode of diarrhea during the study duration of one year was considered an incident case of acute diarrhea. Cumulative incidence was calculated and presented as percentages of incident cases of acute diarrhea out of the total children under observation in one year. Descriptive results were presented as numbers and percentages in a frequency table. Univariate and multivariable logistic regression analyses were utilized to study the association between demographic/built environment characteristics and acute diarrheal disease.

Ethical approval was obtained from the Institutional Ethics Committee (Human), National Institute for Research in Environmental Health (approval number: NIREH/BPL/IEC/2020-21/198 dated June 22, 2020). The study adhered to ethical guidelines, and informed consent was obtained from the parents or guardians of the participating children. Confidentiality and privacy of the participants were strictly maintained throughout the study. Moderate to severely ill cases found during the survey were referred to nearby government facilities.

## Results

Enrolment of the eligible children was conducted from Anganwadis from December 2021 to January 2022. Prospective data collection every month was conducted for one year from February 2022 to February 2023. After applying sampling methods, 658 participants were contacted to participate in the study. Final data was analyzed for 620 participants after excluding 38 participants for various reasons (denied consent: 3; lost to follow-up: 32; moved out of study area: 3).

The most common age group of the participants was one to two years (n = 172, 27.7%), followed by zero to one year (n = 131, 21.1%), three to four years (n = 117, 18.9%), two to three years (n = 116, 18.7%), and four to five years (n = 84, 13.5%). In our study, the number of male (n = 325, 52.4%) participants was slightly higher than female participants (n = 295, 47.6%). Most families did not have a Below Poverty Line (BPL) certificate (n = 440, 71.0%), whereas fewer families had BPL status (n = 180. 29.0%). Most mothers were homemakers (n = 584, 94.2), while very few were working (n = 36, 5.8%). About one-fifth (n = 138, 22.3%) heads of the families were illiterate, while most were educated up to the secondary level of schooling (n = 174, 28.1%), followed by high school (n = 139, 22.4%), primary school (n = 111, 17.9%), graduate and above (n = 38, 6.1%), and senior secondary school (n = 20, 3.2%) (Table 1).

**Table 1:** Descriptive information of the participants. BPL: Participants belonging to families having a Below Poverty Line (BPL) certificate provided by the government of India.

Variables	Variable categories	Number (N= 620)	Percentage (%)
Age (in years)	0–1	131	21.1
	1–2	172	27.7
	2–3	116	18.7
	3–4	117	18.9
	4–5	84	13.5
Sex	Female	295	47.6
	Male	325	52.4
Families having a Below Poverty Line (BPL) certificate	Yes	180	29.0
	No	440	71.0
Occupation of the mother	Working	36	5.8
	Homemaker	584	94.2
Education of the head of the family	Illiterate	138	22.3
	Primary	111	17.9
	Secondary	174	28.1
	High school	139	22.4
	Senior secondary	20	3.2
	Graduate and above	38	6.1
Source of drinking water	Piped	573	92.4
	Other	47	7.6
Method of purification of drinking water	Chlorine tablet/Boiling/Filtering	34	5.5
	Other	586	94.5
Flush latrine in the house	Yes	599	96.6
	No	21	3.4
Open drainage near the house	Yes	97	15.6
	No	523	84.4
Flies in house	Yes	256	41.3
	No	364	58.7

Most (n = 573, 92.4%) of the families had piped water supply in their house, and very few (n = 47, 7.6%) were getting water from outside their house. Very few (n = 34, 5.5%) families were using the recommended water purification method, i.e., chlorine tablet, boiling, or filtering, whereas most (n = 586, 94.5%) of the families used other or no water purification methods in their house. We also found that 15.6% (n = 97) of the houses had open drainage near their house, and 84.4% (n = 523) of the houses had closed systems for drainage. Almost all (n = 599, 96.6%) of the families had flush latrines in their houses, and only a few went outside of their houses for defecation (n = 21, 3.4%). The availability of flush latrines was 100% (299 out of 299) in BSUP colonies compared to 93.5% (300 out of 321) houses in slums (Table 1).

The cumulative incidence of acute diarrheal diseases in the study population was 23.8% (148 out of 620) during the study year, which was distributed almost equally in BSUP (24.4%, 73 out of 299) and slum area (23.4%, 75 out of 321).

On univariate and multivariable logistic regression analyses, we found that most of the variables, i.e., sex (adjusted odds ratio (OR) = 1.15, 95% confidence interval (CI) = 0.79-1.68, p = 0.477), BPL status (adjusted OR = 1.19, 95% CI = 0.78-1.81, p = 0.418), occupation of the mother (adjusted OR = 0.94, 95% CI = 0.41-2.16, p = 0.885), education of the head of the family (adjusted OR = 1.15, 95% CI = 0.73-1.80, p = 0.543), residential area (adjusted OR = 1.04, 95% CI = 0.69-1.58, p = 0.847), piped water source in the house (adjusted OR = 0.85, 95% CI = 0.33-2.17, p = 0.730), method of water purification used (adjusted OR = 1.29, 95% CI = 0.54-3.09, p = 0.563), and open drainage near the house (adjusted OR = 0.50, 95% CI = 0.25-1.01, p = 0.054) did not have any significant association with cumulative incidence of acute diarrheal disease in the study population (Table 2).

**Table 2:** Univariate and multivariable logistic regression results of association between demographics/built environment characteristics and acute diarrheal disease. Model Information: To calculate the adjusted OR, the model was run with all the variables in the table as independent variables and cumulative incidence of diarrheal disease as dependent variables. *: P-value <0.05 was considered statistically significant. OR = odds ratio; CI = confidence interval; BPL = Below Poverty Line; BSUP = Basic Services to the Urban Poor

Variables	Variable categories	Non-adjusted OR (95% CI)	P-value (non-adjusted)	Adjusted OR (95% CI)	P-value (adjusted)
Age	Age in number of years	0.88 (0.77-1.01)	0.074	0.86 (0.75-0.99)	0.041*
Sex	Female	Reference			
	Male	1.09 (0.75-1.6)	0.65	1.15 (0.79-1.68)	0.477
Families having a BPL certificate	No	Reference			
	Yes	1.09 (0.729-1.63)	0.673	1.19 (0.78-1.81)	0.418
Occupation mother	Housemaker	Reference			
	Other	0.91 (0.40-2.03)	0.811	0.94 (0.41-2.16)	0.885
Education of the head of the family	Illiterate	Reference			
	Literate	1.11 (0.72-1.72)	0.641	1.15 (0.73-1.80)	0.543
Residence area	BSUP colony	Reference			
	Slum	0.94 (0.65-1.37)	0.759	1.04 (0.69-1.58)	0.847
Water source	Piped	Reference			
	Other	0.85 (0.41-1.76)	0.665	0.85 (0.33-2.17)	0.73
Method of purification of drinking water	Chlorine tablet/Boiling/Filtration	Reference			
	Other	1.22 (0.52-2.87)	0.645	1.29 (0.54-3.09)	0.563
Open drainage near the house	Yes	Reference			
	No	0.64 (0.36-1.11)	0.113	0.50 (0.25-1.01)	0.054
Flush latrine in the house	Yes	Reference			
	No	3.03 (1.26-7.30)	0.013	4.96 (1.81-13.59)	0.002*

We found that lower age (adjusted OR = 0.86, 95% CI = 0.75-0.99, p = 0.041) and non-availability of flush latrines in the house (adjusted OR = 4.95, 95% CI = 1.80-13.59, p = 0.002) were statistically associated with a higher incidence of acute diarrheal disease among the study population (Table 2).

## Discussion

In this research, we analyzed data from 620 participants. The cumulative incidence of acute diarrheal diseases in the study population was 23.8% (148 out of 620) from February 2022 to February 2023. These results are in line with Ahmed et al. (2008), who reported a 25.5 % prevalence of diarrhea among children under five years old in Kashmir, India. NFHS-5 (2019-2021) reported a comparatively lower prevalence of diarrhea (7.3%), which may be due to the survey’s cross-sectional nature and recall of the last 15 days. Whereas, in our study, we analyzed cumulative data over one year. The observed incidence may be because the study population represented underprivileged communities having poor sanitation and unhygienic infrastructure [22].

On multivariable logistic regression analyses, we found that after adjusting for other variables, lower age and unavailability of flush latrines in the house were significantly associated with the higher cumulative incidence of acute diarrheal disease among the study population. Similar findings have been reported by various original research and reviews published in India and other countries [23-25]. This effect of age on diarrhea incidence may be due to the lack of immunity in lower age groups compared to older ones [23,25].

We also analyzed demographic variables in multivariable logistic regression analyses and found that sex, BPL status, occupation of the mother, education of the head of the family, and residential area had no significant association with the cumulative incidence of acute diarrheal disease among the under-five study population. NFHS-5 found that children from rich households and girls are less likely to suffer from diarrheal disease [26].

Built environment variables such as residential area, piped water source in the house, method of water purification used, and open drainage near the house had no significant association with the cumulative incidence of acute diarrheal disease. However, all these factors were found to be associated with diarrheal disease in previous studies [25,26]. We did not find any significant association with various demographic and built environment factors because the study population belonged to similar sociodemographic and household characteristics. Another reason may be because, in this study, the sample size was calculated to measure the cumulative incidence of diarrhea, not to find the association with risk factors.

In our survey, we covered only the underprivileged population residing in BSUP and slum colonies, which may not be a true representation of the general population. Further research conducted on heterogeneous populations with higher sample sizes might be helpful to shed light on this context.

## Conclusions

In this research, the cumulative incidence of diarrhea was 23.8% among the under-five-year-old population living in underprivileged areas of Bhopal, India. This incidence was significantly associated with lower age and the unavailability of flush latrines in the house. It is worth mentioning that the availability of flush latrines was 100% in BSUP colonies, indicating the importance of this government initiative.
